# Early assessment of acute ischemic stroke in rabbits based on multi-parameter near-field coupling sensing

**DOI:** 10.1186/s12938-022-00991-y

**Published:** 2022-03-27

**Authors:** Gen Li, Shengtong Yin, Man Jian, Jingbo Chen, Lingxi Zeng, Zelin Bai, Wei Zhuang, Bingxin Xu, Shengjie He, Jian Sun, Yujie Chen

**Affiliations:** 1 School of Pharmacy and Bioengineering, Chongqing University of Technology, Chongqing, China; 2 College of Biomedical Engineering, Army Medical University, Chongqing, China; 3 Department of Neurosurgery, Southwest Hospital, Army Medical University, Chongqing, China

**Keywords:** Acute ischemic stroke, Near-field coupling, Multi-parameter continuous assessment, Bilateral common carotid artery ligation

## Abstract

**Background:**

Early diagnosis and continuous monitoring are the key to emergency treatment and intensive care of patients with acute ischemic stroke (AIS). Nevertheless, there has not been a fully accepted method targeting continuous assessment of AIS in clinical.

**Methods:**

Near-field coupling (NFC) sensing can obtain the conductivity related to the volume of intracranial components with advantages of non-invasiveness, strong penetrability and real-time monitoring. In this work, we built a multi-parameter monitoring system that is able to measure changes of phase and amplitude in the process of electromagnetic wave (EW) reflection and transmission. For investigating its feasibility in AIS detection, 16 rabbits were chosen to establish AIS models by bilateral common carotid artery ligation and then were enrolled for monitoring experiments.

**Results:**

During the 6 h after AIS, the reflection amplitude (RA) shows a decline trend with a range of 0.69 dB and reflection phase (RP) has an increased variation of 6.48° . Meanwhile, transmission amplitude (TA) and transmission phase (TP) decrease 2.14 dB and 24.29° , respectively. The statistical analysis illustrates that before ligation, 3 h after ligation and 6 h after ligation can be effectively distinguished by the four parameters individually. When all those parameters are regarded as recognition features in back propagation (BP) network, the classification accuracy of the three different periods reaches almost 100%.

**Conclusion:**

These results prove the feasibility of multi-parameter NFC sensing to assess AIS, which is promised to become an outstanding point-of-care testing method in the future.

## Background

Stroke is the leading cause of long-term disability in developed countries and one of the top causes of mortality worldwide. As reported by American Stroke Association, acute ischemic stroke (AIS) is responsible for almost 90% of all strokes. According to the Guidelines for the Early Management of Patients with Acute Ischemic Stroke (AHA/ASA, 2019), AIS patients meeting the screening criteria benefit from intravenous thrombolysis within 3 h [1]. A multi-center retrospective study reported that precise characterization of intracranial blood supply status is required to select suitable treatment methods for cerebral arterial recanalization and tissue reperfusion, which predicts good functional outcomes following AIS [2]. Therefore, early diagnosis and continuous monitoring are the key to emergency treatment and intensive care of patients with AIS.

Owing to the advantages of no secondary pain and wide clinical applicability, the non-invasive detection has developed rapidly in the past decade, but there has been no fully dependable method of early diagnosis and continuous monitoring of AIS. The diagnosis of AIS in clinical mainly relies on imaging methods such as computed tomography (CT), magnetic resonance imaging (MRI), positron emission tomography, etc. However, this type of equipment is bulky and generally fixed and their low time resolution leaves a risk of delay in treatment. Although mobile stroke units based on CT or MRI have promising prospects, the burden of using the units are high for most hospitals worldwide, especially in some developing and underdeveloped countries. Transcranial Doppler (TCD) often used for intermittent monitoring utilizes cerebral blood flow velocity (CBFV) for dynamic assessment of AIS. Sympathetic nerve stimulation or infusion of vasoactive drugs may cause changes in the diameter of cerebral arteries, making it difficult to measure CBFV accurately [3]. Furthermore, it cannot detect the flow velocity in intracranial small vessels and capillaries. It is proved that intracranial small vessels and capillaries play a vital role in the cerebral blood flow (CBF) regulation. The laser Doppler flowmeter (LDF) can also measure CBFV, but its detection depth is limited and the measurement results are easily interfered by environmental factors [4]. Near-infrared spectroscopy (NIRS) measures the changes of blood oxygen and deoxyhemoglobin in blood vessels, by which it can realize continuous and non-invasive monitoring of AIS. The prerequisite is that the amount of light scattering remains unchanged and changes in attenuation are only caused by absorption [5]. However, its detection depth is also limited and the composition of the intracranial tissues will change significantly after AIS, resulting in changes in both light absorption and scattering. Microwave imaging can reflect the pathophysiological activities of biological tissues based on non-invasive detection of relative permittivity. There exists a difference in dielectric properties between blood and gray matter, thus the relative permittivity of ischemic tissues alters compared to normal brain tissues. Meanwhile, some theoretical derivation and model-based experiments have proved the potential of stroke imaging by microwave technology [6–8]. Therefore, microwave imaging can theoretically realize non-invasive and continuous monitoring of AIS. But its penetration depth is limited and the detection accuracy is greatly affected by background noise. The bioelectrical impedance (BEI) method uses electrodes to inject current into the cranium and measures the change of boundary potential to detect AIS. However, it is difficult to guarantee a stable electrical contact between the electrodes and scalp in practice, and the attenuation of injection current and poor penetration caused by the high resistivity of the skull affect the accuracy [9].

As an emerging biomedical electromagnetic detection method in recent years, near-field coupling (NFC) sensing focuses on the coupling effect of electromagnetic wave (EW) with the surface and internal tissues of living organisms, which has advantages of non-invasive, strong penetrability and real-time continuous monitoring. The physiological and pathological information of tissues will modify the amplitude/phase of electromagnetic waves. When the NFC sensor emits the EW signal to the head, a portion of EW is directly reflected, and the rest is transmitted to deep area of brain through skull, forming two signals with different paths. They are defined as reflection signal and transmission signal. Previous researches have shown that the amplitude and phase shift relative to the emission signal are related to the conductivity of the whole brain [10, 11]. Oziel et al. utilized the Z-parameter to monitor blood accumulation in the head [12]. Their latest research further has shown that the change of amplitude/phase increases or decreases or even shows a non-linear trend with the change of injected blood volume at different measurement frequencies [13–15]. Griffith et al. developed a passive skin patch sensor for the non-invasive detection of brain volume. The physical simulation experiments and human trials results showed that this sensor can monitor cranial fluid volume changes, significant for auxiliary diagnosis of stroke, cerebral concussion and intracranial pressure monitoring [16]. Saied et al. proposed a wearable detection system based on flexible fabric materials for neurodegenerative diseases. The sensor consists of six monopole planar antennas with two independent resonance frequencies (800 MHz and 2.1 GHz). In the brain atrophy and unilateral ventricle expansion simulation experiments, the magnitude–frequency characteristics of the reflection and transmission parameters show different trends, indicating that the system has the potential to distinguish different neurodegenerative diseases [17]. McDermott et al. proposed a dual-frequency symmetrical difference method for stroke diagnosis and verified its feasibility by simulating normal and hemorrhage and blood clot lesions on a 4-layer finite element method head model [18]. Jiang et al. used a cylindrical waveguide as a self-resonant cavity sensor to build a detection system with a working frequency band (FB) of 100–400 MHz and detected the rate and volume of hematoma enlargement on a 3D head model made by a stereolithography machine [19]. The results showed that 1 cm^3^ bleeding can be detected in TE111 mode.

Our research team has long been committed to the detection of cerebrovascular diseases based on NFC sensing. We built a real-time continuous brain edema monitoring system based on the frequency-dependent conductivity of biological tissues and the NFC theory and performed 24-h monitoring experiments in rabbits, which proved its feasibility in brain edema monitoring [20, 21]. Furthermore, we demonstrated the relationship between the NFC signal and the two common parameters of neurosurgery intensive care and established NFC monitoring models of the main pathophysiological response after brain injury, combined with machine learning methods [22, 23]. Further, a clinical trial was performed in the Department of Neurosurgery, Southwest Hospital, Chongqing, China. The result indicates that the NFC sensing is able to monitor the intracranial pathophysiological change after basal ganglia hemorrhage in real-time [24]. In 2020, we carried out an experimental study on NFC detection of AIS established by the carotid artery ligation in rabbits. The results show that the NFC signal has obvious changes after AIS and the signal shows significant difference between two degrees of carotid artery ligation models, preliminarily proving the feasibility of NFC method in detecting AIS [25]. Previous research only selected the phase of the transmission signal as the detection parameter, thus resulting in the poor measurement consistency. More importantly, it is difficult to monitor the dynamic changes of the intracranial pathophysiological state after AIS accurately. In addition, our previous studies have shown that using the features from both the reflection and the transmission signals can classify three different states after traumatic brain injury with 100% accuracy, which seems to play an important role in improving the reliability of monitoring AIS by NFC sensing [26].

In this study, we established a multiple-parameter NFC monitoring system for AIS by making use of the reflection and transmission characteristics of the two-port test network. In the 6-h AIS monitoring experiment of 16 rabbits, the amplitude and phase spectra of reflection and transmission signals in the FB (300 kHz200 MHz) were obtained at a rate of 1 h. After analyzing the phase and amplitude information of reflection and transmission signals jointly, the optimal frequency for AIS detection was found. Based on the changes in reflection amplitude (RA), reflection phase (RP), transmission amplitude (TA) and transmission phase (TP) at the optimal frequency, we investigated the effectiveness of monitoring intracranial states dynamically in the development of AIS. Finally, via back propagation (BP) neural network, the classification of different intracranial states before and after AIS was performed based on the monitoring data of the four parameters. By using the four parameters, respectively, vs altogether as identification features, the accuracy of the classification was compared.

## Results

### Frequency selection for AIS detection

**Fig. 1:**
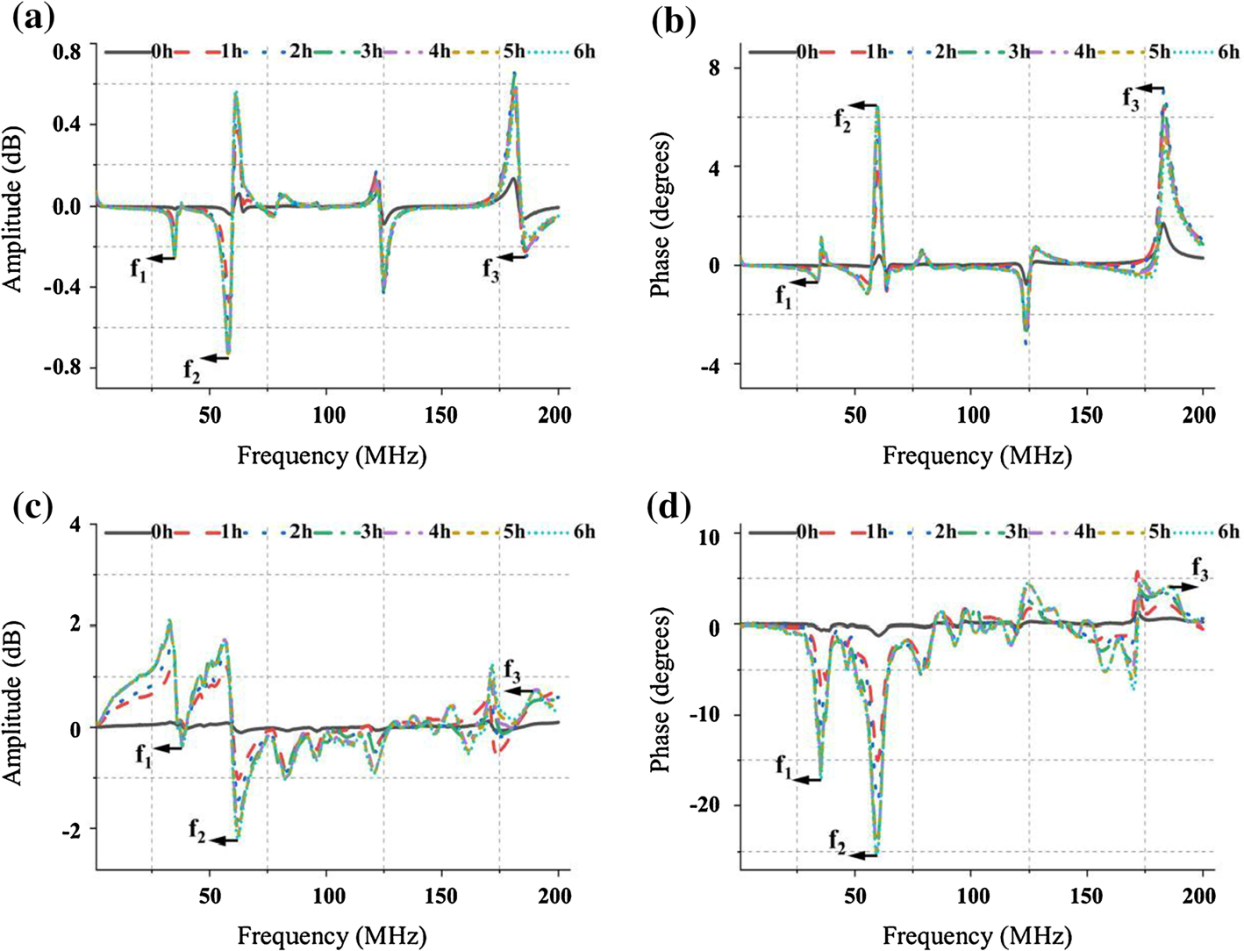
The 6-h mean changes (*n* = 16) of reflection amplitude (RA), reflection phase (RP), transmission amplitude (TA) and transmission phase (TP) in AIS monitoring experiment. The frequency range is from 300 kHz to 200 MHz. **a** is RA and **b** represents RP. The results of TA and TP are shown in **c** and **d**, respectively

The mean changes (*n* = 16) of RA, TA, RP and TP in 6 h are shown in Fig. 1. With the increase of AIS severity, the four parameters all have large fluctuations at some frequency points in the sweeping band (300 kHz200 MHz). However, RA (Fig. 1a) and RP (Fig. 1b) almost have no large fluctuations over time at those frequency points with poor impedance matching, which shows strong stability compared with TA (Fig. 1c) and TP (Fig. 1d). Meanwhile, TA and TP hold a larger variation range at the frequency points (f_1_, f_2_ and f_3_) that have excellent impedance matching than RA and RP, that is, the amplitude and phase of the transmission coefficient is more sensitive than reflection coefficient. This result is consistent with the two-port network test principle and previous researches, proving that the transmission coefficient has more influencing factors than reflection coefficient, and its variation range caused by the change in conductivity of the target under test (TUT) is greater.

**Fig. 2:**
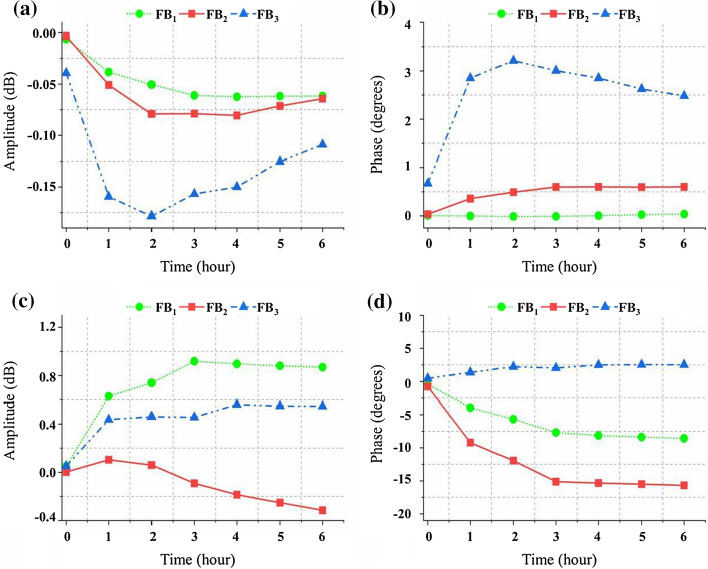
The average variations of the four monitoring parameters within 6 h in FB_1_ (35.622 ± 5 MHz), FB_2_ (59.836 ± 5 MHz) and FB_3_ (189.141 ± 5 MHz). **a** is RA and **b** represents RP. The results of TA and TP are shown in **c** and **d**, respectively. The variation trends of monitoring parameters over time in different frequency bands are drawn by three colors (green, red and blue)

The average variations of the four monitoring parameters within 6 h in FB_1_, FB_2_ and FB_3_ are shown in Fig. 2. As shown in Fig. 2a, the RA in FB_1_ and FB_2_ generally show a gradual downward trend, while in FB_3_ it exhibits a large range of oscillations reflecting poor stability. The RP in FB_2_ shows a gradual upward trend in Fig. 2b, while it has no obvious changes in FB_1_ and fluctuates around the initial value with a large range in FB_3_. In Fig. 2c, the TA in FB_1_ first rises and then gradually decreases. It is more sensitive than that in FB_2_ and FB_3_. Figure 2d depicts that the TP has a gradual downward trend over time in FB_1_ and FB_2_ but almost remains unchanged in FB_3_. According to the analysis of the spectrum data of the four parameters from 300 kHz to 200 MHz, the reflection parameters are more stable than the transmission parameters, and the transmission parameters are more sensitive than the reflection parameters. The variation ranges of the four parameters in FB_3_ are inconsistent with the analysis of the spectrum data, indicating that most of the EW signals sent by port 1 are lost. Under such mismatching condition, RA, TA, RP and TP were greatly influenced by external interference. FB_1_ and FB_2_ have similar performance, but only the TA of FB_1_ changed slightly higher than that of FB_2_. And the changes of RA, RP and TP of FB_1_ are less than those of FB_2_. Especially, the RP of FB_1_ remains almost constant from 0 to 6 h. These results indicate that FB_2_ and f_2_ are the optimal FB and point suitable for AIS detection.

### Multiple-parameter monitoring outcomes

**Fig. 3:**
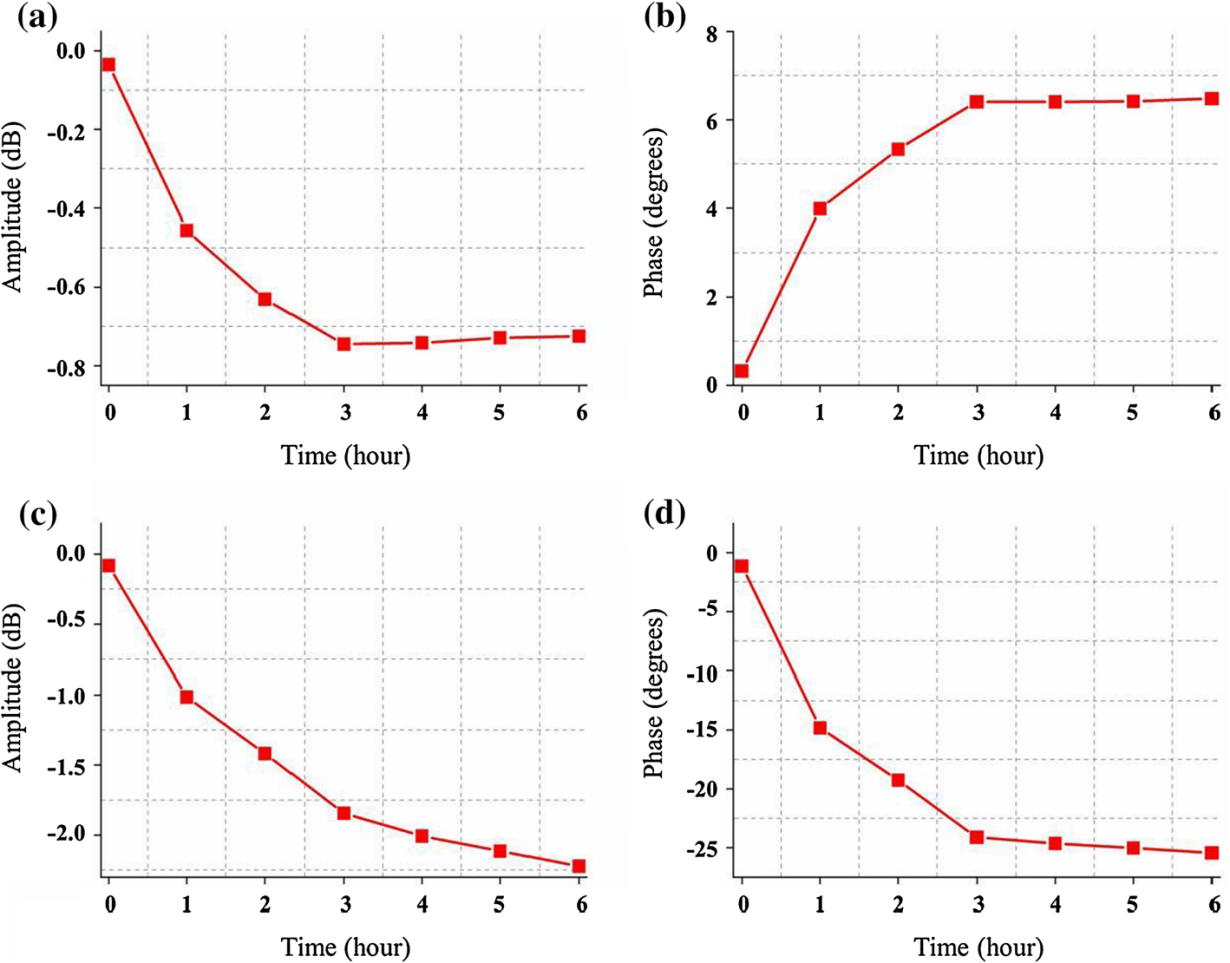
The change trend of reflection amplitude (RA), reflection phase (RP), transmission amplitude (TA) and transmission phase (TP) in 6 h after bilateral carotid artery ligation at f_2_ (59.836 ± 0.179 MHz). **a** is RA and **b** represents RP. The results of TA and TP are shown in **c** and **d**, respectively

The change trends of the four monitoring parameters in 6 h after bilateral carotid artery ligation at f_2_ are shown in Fig. 3. The arterial blood supply system of the brain tissue is composed of four major arteries, namely the carotid arteries and the vertebral-basal arteries. Among them, 60% of the blood supply is completed by the carotid arteries. When the rabbits suffer from bilateral carotid artery ligation, the blood volume in the skull decreases rapidly, resulting in a drop in the overall conductivity of the brain. As shown in Fig. 3 (a), (c), (d), RA, TA and TP show obvious downward trends in the first 3 h, which are consistent with the rapid decrease in the overall conductivity of the brain caused by early AIS. However, the RP shows an upward trend in the first 3 h as shown in Fig. 3 (b), consistent with the previous research [25]. Although the bilateral carotid arteries have been ligated, the vertebral-basal arteries still supply blood to the brain tissue, compensating cerebral blood volume through vasodilation and accelerating blood flow velocity several hours after bilateral carotid artery ligation [26]. Compared to the previous period, the rate of AIS slows down. Figure 3 shows that the decline rate of these four monitoring parameters decreased significantly after 3 h of bilateral carotid artery ligation. Especially, RA, RP and TP tend to be flat. These results are consistent with the mechanism of ischemia caused by the common carotid artery ligation and justify the selection of the sampling interval (1 h) this study during such a slow ischemic process. To summarize, the multi-parameter NFC can reflect the pathophysiological process of AIS.

**Fig. 4:**
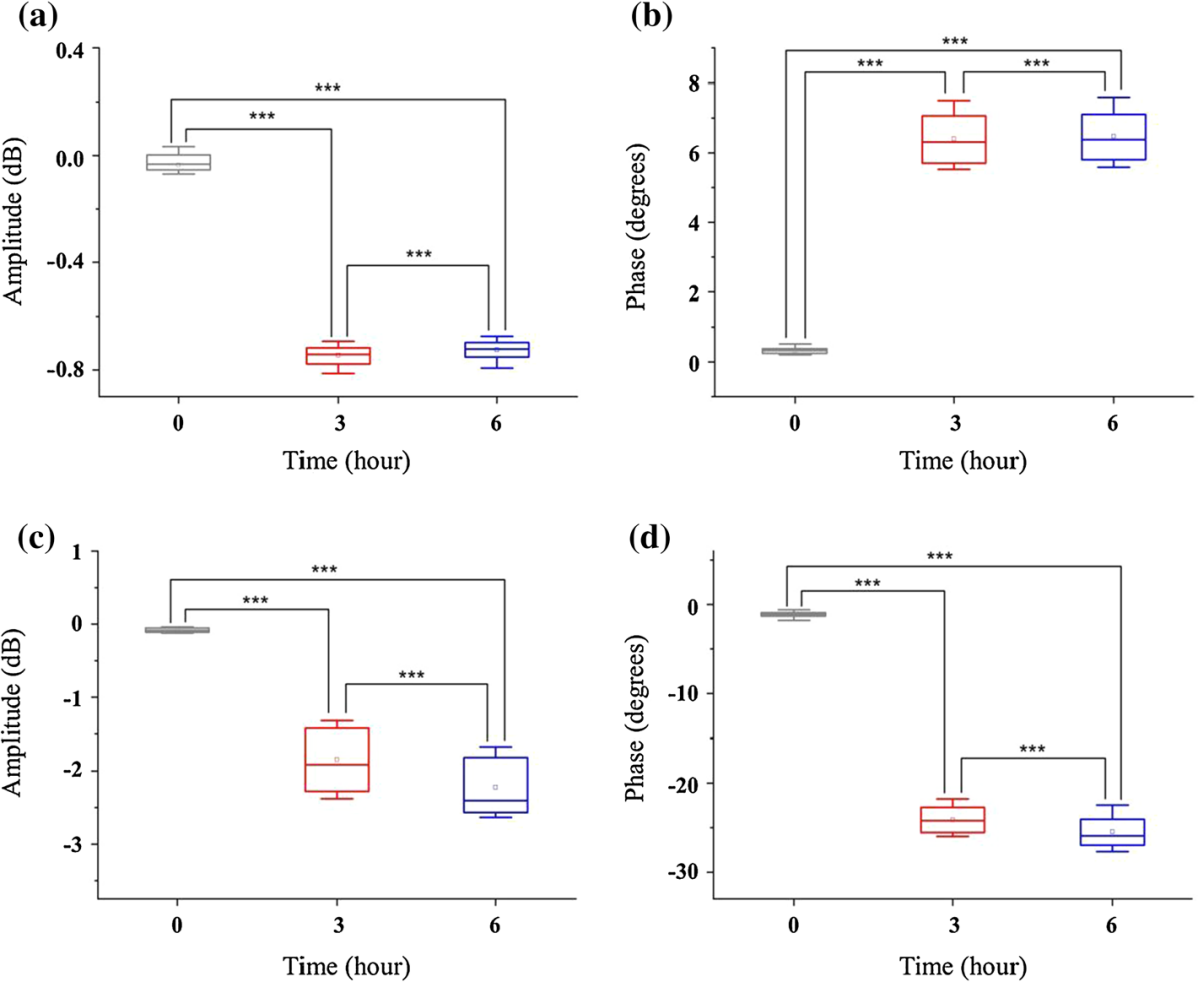
The paired sample *t* test (*α* = 0.05) results of reflection amplitude (RA), reflection phase (RP), transmission amplitude (TA) and transmission phase (TP) among three time points (before bilateral carotid artery ligation, 3 h after ligation and 6 h after ligation). **a** is RA and **b** represents RP. The results of TA and TP are shown in **c** and **d**. The number of symbol (*) reflects the size of *P* value. Symbol (***) indicates extremely significant (*P* < 0.001)

Figure 4 depicts the paired sample *t* test (*α* = 0.05) results of the four monitoring parameters among three time points (before bilateral carotid artery ligation, 3 h after ligation and 6 h after ligation). The four monitoring parameters demonstrate extremely significant differences in these three points before and after ligation, indicating that multi-parameter NFC can effectively distinguish different intracranial pathophysiological states of AIS.

### Classification of different intracranial pathophysiological states

**Fig. 5:**
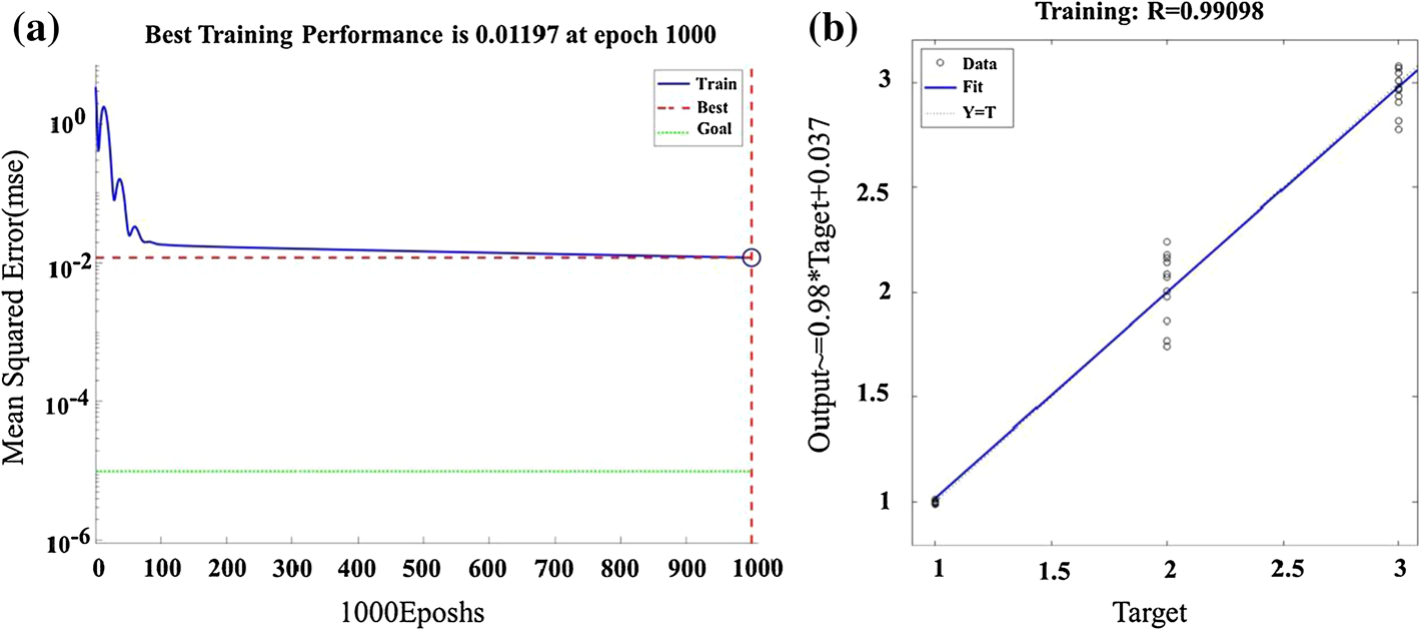
The classification training of three different intracranial pathophysiological states (1, 2, 3) using BP algorithm. The training target was set to 0.00001. **a** Represents the change trend of mean squared error with increasing number of epochs. **b** Depicts regression analysis of the BP algorithm. The *R* value is 0.99098

The three different intracranial pathophysiological states (1, 2, 3) were classified by the BP network. As shown in Fig. 5(a), the number of training sessions reached 1000 times and took 4 s under ordinary PC. As the number of training increases, the mean square error gradually decreases, indicating that the training gradually converges. Figure 5 (b) depicts that the *R* value is 0.99098 in the given fitting equation, showing that the fitting effect is excellent.

**Fig. 6:**
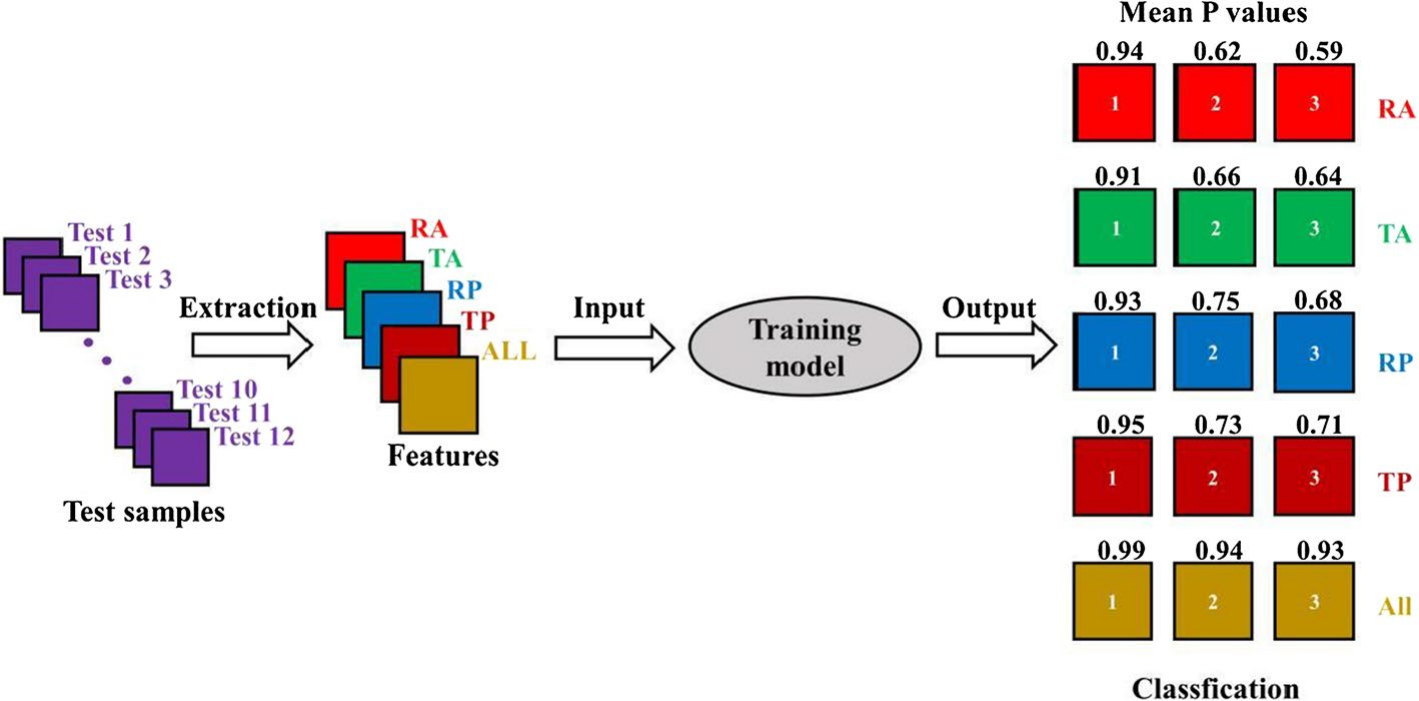
The classification tests of three different severity levels (1, 2, and 3) after training. Four groups were randomly set as test samples (*n* = 4 × 3 = 12). The four parameters were used individually and all as recognition features. Then they were input into the training model in turn. The mean *P* values were calculated by the outputs the training model (*n* = 4), which depicts the classification accuracy, ranging from 0 to 1. Larger mean *P* value corresponds to higher accuracy

The remaining four groups were set as test samples (*n* = 12) and the test results are shown in Fig. 6. When the four monitoring parameters were individually used as identification features, the output *P* value of state 1 ranges from 0.91 to 0.95. However, the output *P* value of state 2 ranges from 0.62 to 0.75 and those of state 3 ranges from 0.59 to 0.71. It illustrates that the RA, TA, RP or TP can classify normal and AIS states with high accuracy, but the performance for the state differentiation after AIS is poor. When they were all used as identification features, the output *P* values of the three different states reach 0.99, 0.94 and 0.93. In other words, the four monitoring parameters are all able to reflect the changes at different stages of AIS, but in the state discrimination of AIS, it is better to use all the parameters as the recognition features, which indicates that using the multi-parameter NFC can achieve a more effective classification performance.

## Discussion

Although intravenous thrombolysis, mechanical thrombus removal from blood vessels and antiplatelet drugs are effective methods for the treatment of AIS, there is an urgent need to develop a safe and reliable diagnostic method to achieve individualized and precise management. The golden time window of thrombolytic therapy for AIS is less than 3 h [27]. In addition, thrombolysis within 3 to 4.5 h can also achieve good therapeutic effect in most cases. However, once it exceeds 4.5 h, both thrombolytic conditions and therapeutic effect will be greatly limited [28, 29]. The current clinical imaging equipment is huge, not suitable for pre-hospital diagnosis and real-time continuous monitoring of patients after admission. Some other non-invasive methods, such as TCD, LDF, NIRS and BEI, have inherent defects in detection depth, continuous monitoring or easy operation. NFC sensing has advantages such as non-invasiveness, strong penetrating, and small size. Chen et al. demonstrated the quantitative relationship between the variation range of the NFC signal and the volume change of intracranial components combining the MRI images [30]. The volume of CBF will decrease obviously after AIS. In addition, there is evidence that cerebral hemorrhage and AIS have different modes of influence on NFC signals [31]. Therefore, the early AIS detection method based on the development of near-field coupling technology meets the current clinical needs and has the theoretical feasibility. Our previous 24 h monitoring experimental study on focal ischemia of rabbits based on NFC shows that the time of infarct caused by local ischemia is about 5–6 h [32]. Combined with the time window of thrombolytic therapy, we carried out an experimental study of AIS monitoring within a 6 h time window based on multi-parameter near-field coupled sensing in this work. Stroke usually leads to hemorrhage and ischemia, the treatments for them are different, so mishandling brings catastrophic consequences. The multi-parameter NFC sensing obtains more comprehensive information about intracranial pathophysiological states than traditional methods, which recognizes ischemia with a high accuracy. Besides, the multi-parameter NFC sensing proposed in this work can continuously and dynamically evaluate the perfusion state of cerebral blood flow after the effective intervention of AIS, which helps clinicians to timely discover secondary pathophysiological reactions such as cerebral edema and encephalomalacia.

The broadband frequency sweeping analysis and time difference offsetting were used to reduce the environmental effects in this work, improving the performance on detection of AIS based on NFC technology. The influences of environmental interference on the NFC detection at different frequencies are inconsistent. Due to the limitation of the selection of excitation signal frequency and the inherent weak conductivity of biological tissues, NFC perform poorly in actual detection. The traditional common methods are to use a single or several limited frequencies, which is difficult to accurately obtain the one with optimal detection performance [33, 34]. Meanwhile, some reported studies of the research groups worldwide have made progress in improving the radiation intensity and uniformity of the near field, providing a vital reference in coil sensor design [35–37]. However, these proposed methods are only suitable for pure electromagnetic simulation calculation and physical experiment to investigate the performance of NFC detection. The characteristics of the TUT itself are ignored and there is a huge gap with the actual situation, resulting in a limited increase of sensitivity. Although biological tissues have weaker conductivity than good conductors such as metals, coupling into the two-port test network will also cause a slight change in the frequency response. Therefore, three feature bands with a width of 10 MHz were selected according to the impedance matching performance of multi-parameter NFC sensing system with the rabbit AIS model in this work. Then, the environmental effects on the NFC detection based on the spectrum with wide FB (300 kHz–200 MHz) were observed. Finally, the ischemic stroke detection band with optimal performance was obtained by analyzing and comparing the average changes in the three feature bands. The influence of environmental noise interference is relatively weak on this band, which increases the sensitivity for detecting intracranial pathophysiological changes. In addition, the NFC spectrum data of anesthetized rabbits were regarded as the reference values including the interested signals, interference from other physiological activities and environmental noises. The reference values were subtracted by the NFC spectrum data collected before and after ligation. Then, we observed and analyzed the results after time difference offsetting to isolate the effect of the brain variation. In order to facilitate the comparison of the state before and after ischemia, the rabbits before common carotid artery ligation were regarded as the control group. And the multi-parameter NFC data of anesthetized rabbits without no other surgical operations were collected as referenced values. In the next work, we will try to select another group of rabbits as the control group and measure their multi-parameter near-field coupling spectra from 0 to 6 h as the reference values. The difference at the corresponding time phase between the reference values and the detection results of the experimental group may include less interference. But this method may also introduce more uncertain variables.﻿

The frequency selection method proposed in this work can quickly determine the optimal excitation frequency so as to continuously collect high-performance NFC signals to achieve monitoring AIS in real-time. The frequency spectrum data based on multi-parameter near-field coupling sensing were collected in the band of 300 kHz to 200 MHz. And the more frequency points, the higher the frequency resolution. If fewer frequency points are selected, the feature band and the optimal detection frequency point of AIS will shift accordingly. This shift affects the sensitivity and stability of multi-parameter NFC sensing in detecting AIS. The determine method of feature band and optimal detection frequency point was proposed and verified in this study. After completing the sweeping with a high frequency resolution and confirming the feature bands, it is possible to obtain superior detection sensitivity and stability by using less frequency points based on continuously sweeping near the bands and reading the multi-parameter NFC sensing data. It took less than one second to obtain a detection result based on the multi-parameter NFC sensing system established in this work. If the number of sweeping points is reduced, the time will be further improved. Therefore, the determination method of feature band and optimal detection frequency point is conducive to the dynamic monitoring of multiple NFC parameters at a higher sampling rate and obtain more detailed information about AIS.

Multi-parameter NFC sensing based on RA, TA, RP and TP is able to monitor the changes dynamically in the intracranial pathophysiological state after AIS more accurately. Similar to near-infrared light, EW will be reflected and transmitted when passing through the brain, but the traditional NFC detection focuses on the transmission parameters and ignores the reflection ones, the reason of which is that the transmission parameters contain more information about the changes of the internal conductivity of the TUT than that of the transmission parameters. In addition, sufficient research evidence shows that the phase is more sensitive than the amplitude but the stability is poorer, which is consistent with the broadband spectrum results on the AIS model in this study [38]. So, TP is often used as a single parameter to detect cerebrovascular diseases, and it is difficult to monitor the complicated intracranial pathophysiological development after AIS. After AIS, the cerebral blood flow (CBF) decreases rapidly, and the CBF regulation will gradually intervene with the increase of time through cerebral arterial vasodilation and intravascular flow velocity rising [39]. According to the conductivity of biological tissues, the cerebrospinal fluid is the highest among intracranial contents, followed by the CBF, and the brain parenchyma is the lowest [40]. From the occurrence of AIS to the loss of compensation, the NFC signal theoretically first declines or rises and then gradually stabilizes. In previous work, we measured TP only on the AIS model established by unilateral and bilateral common carotid artery ligation. And the laser Doppler cerebral blood flow velocity (CBFV) was regarded as a reference. The monitoring outcomes showed that both TP and laser Doppler CBFV have continuous downward trends over time. Statistical analysis has illustrated that there is a correlation between them and TP can distinguish different ligation methods. Laser Doppler CBFV is mostly related to the focal blood supply from the major blood vessels and its downward trend proves the occurrence of ischemia to a certain extent. But the TP reflecting changes in the volume of the global CBF does not match the overall intracranial conductivity at different periods after AIS. Indeed, it is difficult for a single NFC parameter to reflect the complex pathophysiological process of AIS accurately, therefore, we proposed multi-parameter NFC sensing in this work. We conducted an experimental study on an animal model established by bilateral carotid artery occlusion causing more rapid injury in order to study the feasibility of multi-parameter NFC Sensing for early assessment of AIS and to facilitate comparison with the previous experimental work. The slopes of the four NFC parameters in this study within 6 h after the occurrence of AIS showed different degrees of decrease over time, which is consistent with the physiological process in which the intracranial blood volume decreases rapidly in the early stage of AIS and then stabilizes with vascular autoregulation (VA) compensation. Clinical studies show that VA compensation has individual differences and has a reliable correlation with the prognosis of AIS. In addition, timely effective intervention before the loss of VA compensation can effectively reduce the incidence of irreversible neurological damage, so it is potential for multi-parameter NFC sensing to be applied to the personalized management and early warning assessment of irreversible neurological damage for AIS patients with different VA compensation levels. With the continuous development of AIS, we infer that the irreversible infarction is gradually forming, the available space for CBF reduces with the expansion of infarction area, and the absolute values of the change in the four NFC parameters will further increase.

The BP network is one of the most widely used artificial neural networks. To enhance the superiority and universality of multi-parameter NFC sensing, we selected it for classification of ischemia at different degrees. When the proposed four parameters were all used as identification features together, three different intracranial pathophysiological states were accurately identified. It is of great significance for clinicians to take effective treatments of AIS in time and to prevent poor prognosis. The four parameters all had obvious upward or downward trends in the 6 h monitoring of AIS. The statistical analysis of differences was used for feature selection, and the results illustrated that they could effectively differentiate the three different states. Therefore, we regarded them as inputs for model training based on BP neural network. By changing the weight and threshold of the internal connection, the minimum error between the output value and the target value is achieved, thereby achieving the purpose of modeling. In this work, the established training model has fast convergence speed and good fitting performance, which meets the expected requirements. The differences in compensatory ability, depth of anesthesia and physical conditions make the same parameters of rabbits change differently at the optimal frequency [41]. Therefore, when the four parameters are used as identification features alone, the classification performance of three different states is poor. When they are all used as identification features, the accuracy of classification reaches almost 100%, which strengthens the value of multi-parameter NFC sensing.

In practical terms, the intracranial pathophysiological process after AIS is very complex. For comparison, this work only verified the feasibility of multi-parameter NFC sensing to monitor AIS in early stage. We will further explore the relationship between the intracranial pathophysiological response after the formation of irreversible infarcts and the proposed four monitoring parameters, through which we may establish the corresponding multi-parameter NFC model. Also, the traditional single excitation-single receiving coils are still used to show the superiority of multi-parameter near-field coupled sensing more directly. Based on the brain volume of human and the practical requirements for neurosurgical monitoring, we will study a new near-field coupling sensor that detect the CBF reduction of left and right hemispheres, respectively, to further improve the accuracy and sensitivity. Combined with the multi-parameter NFC sensing, a clinical trial study of evaluating AIS will be carried out. The BP network has some shortcomings, for example, the initial weights and thresholds are created artificially, which may lead to bias in the model [42]. In addition, the selection of samples is critical to the quality of the training model. In the next step, we will improve the algorithm based on expanding the sample size and comparison with other algorithms.

## Conclusion

This work established a multi-parameter NFC sensing system of AIS based on the reflection and transmission characteristics of the two-port test network, which has advantages of non-invasiveness, non-contact, good portability, and real-time continuous monitoring. Combined the impedance matching, three feature frequency points were found through joint analysis of reflection and transmission amplitude spectrum. By analyzing the changes of RA, TA, RP and TP in the sweeping band and comparing the average variations around the feature frequency, we proved that the optimal detection frequency of AIS is among the three points. During the 6 h after bilateral carotid artery ligation in rabbits, the RA, TA, RP and TP at the optimal detection frequency first change rapidly and then gradually stabilized, indicating the feasibility of monitoring the intracranial pathophysiological development in early stage of AIS. When the four parameters were all used as identification features, the classification accuracy of the three different intracranial states reached almost 100%. This study provides a new solution to early detection of AIS, and lays the foundation for the next step to predict large intracranial infarction accurately and to propose a personalized clinical monitoring plan.

## Methods and materials

### Principle of near-field coupling detection

**Fig. 7:**
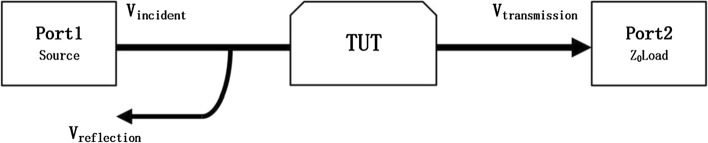
Two-port test principle containing a signal source and a TUT. An incident signal (${V}_{incident}$) is added with Port 1

Figure 7 depicts a two-port test system containing a signal source and a target under test (TUT). The relationship between input and output signals are defined by using incident signals, reflection signals and transmission signals. When Port 1 is added with an incident signal and Port 2 has no input, some of the incident signal is reflected due to the mismatch of two-port network, that is, the reflection signal, and the rests are transmitted to Port 2 through the TUT, the transmission signal.

${V}_{reflection}$ is the reflection signal and ${V}_{transmission}$ is the transmission signal. The two signals have different paths in the two-port test system and the total size of them is theoretically equal to that of ${V}_{incident}$.

The reflection coefficient refers to the relationship between the reflection signal and the incident signal is defined as:

1 $$Reflection\,coefficient=\frac{{V}_{reflection}}{{V}_{incident}}=\rho \mathrm{\angle }\theta .$$

The transmission coefficient refers to the relationship between the transmission signal and the incident signal is defined as:

2 $$Transmission\,coefficient=\frac{{V}_{transmission}}{{V}_{incident}}=\tau \mathrm{\angle }\varphi ,$$

where $\rho$ and $\tau$ are the amplitudes of reflection coefficient and transmission coefficient, respectively. $\theta$ and $\varphi$ represent the phase of reflection coefficient and transmission coefficient, respectively.

The four parameters are all related to the conductivity of the TUT. Our previous work has proved that $\theta$ and $\varphi$ are related to the conductivity of the TUT [26]. In this study, $\theta$ and $\varphi$ are defined as RP and TP. Similarly, $\rho$ and $\tau$ are defined as RA and TA, respectively. In practical circuits, parts of the incident signals are transmitted from Port 1 to Port 2 via TUT, some are directly reflected back due to mismatch, and the rests are lost in the transmission process [43]. Therefore, $\rho$ and $\tau$ largely depend on the impedance matching degree of the entire network, which is modified by the conductivity of the TUT.

### Multi-parameter near-field coupling sensing system

The multi-parameter near-field coupling sensing system mainly includes a portable vector network analyzer (Copper Mountain, M5065), a coil sensor suitable for animal experiments, and real-time monitoring software for AIS.

The coil sensor consists of excitation coil and receiving coil, wounded at both ends of the plexiglass tube by use of 12 turns of AWG32 copper wire. According to the skull size of the rabbits, the radius of the coils was customized as *R*1 = *R*2 = 5.4 cm, the distance was 12 cm [44].

The vector network analyzer connected to a notebook computer by USB line is the hardware core of the entire system. It connects the coil sensor via coaxial line, which is responsible for providing an excitation source and transmitting a near field to the TUT through the excitation coil and at the same time, the transmission signal from receiving coil is collected, finally, obtaining the NFC measurement results after completing the signal processing and phase calculation.

The real-time monitoring software for AIS, as the control and function core of the entire system, is compiled on the host computer and then called by vector network analyzer, which mainly realizes the functions of automatic parameter setting and NFC data collection in real-time. The parameters are listed in Table 1. The intermediate frequency band width (IFBW) setting generally needs to balance two factors: dynamic range and measurement speed. The overall guideline is to use a wide IFBW as far as possible while ensuring the requirements of dynamic range and trace noise. When IFBW was set to 3 kHz, dynamic range was about 60 dB, sweeping time was less than 1 s, and the trace noise magnitude was 0.002 dB rms. Besides, the noise floor of the VNA is  130 dBm/Hz.

**Table 1:** Measurement parameters of multi-parameter NFC for AIS

Name of parameters	Setting
Sweeping band	300 kHz to 200 MHz
Sweeping points	1601
IFBW	3 kHz
Output power	10 dBm
Trigger source	Internal
Trigger mode	Continuous
Sampling interval	1 h
Data format	RA, TA, RP and TP

### Experimental design

For investigating the feasibility of warning and monitoring AIS based on multi-parameter near-field coupling, sixteen healthy rabbits (2.0–2.6 kg, available from the Laboratory Animal Center of Army Medical University, marked No.1 to No.16) were enrolled to carry out the AIS monitoring experiment. The experiment was carried out in accordance with the Helsinki Declaration and IASP guidelines, respecting animal life, reducing animal stress, pain and injury, and euthanasia was adopted after the experiment.

The common carotid artery ligation method was used to prepare the AIS model [45]. Rabbits were first injected with urethane (25%, 5 ml) via ear vein for anesthesia. Then the hairs on their neck and head were removed. After disinfection, the neck skin cutting in the center and the blunt separation were operated to expose and separate the bilateral common carotid arteries. Finally, the surgical ligation line was fastened to block the common carotid arteries bilaterally for the experiment.

**Fig. 8:**
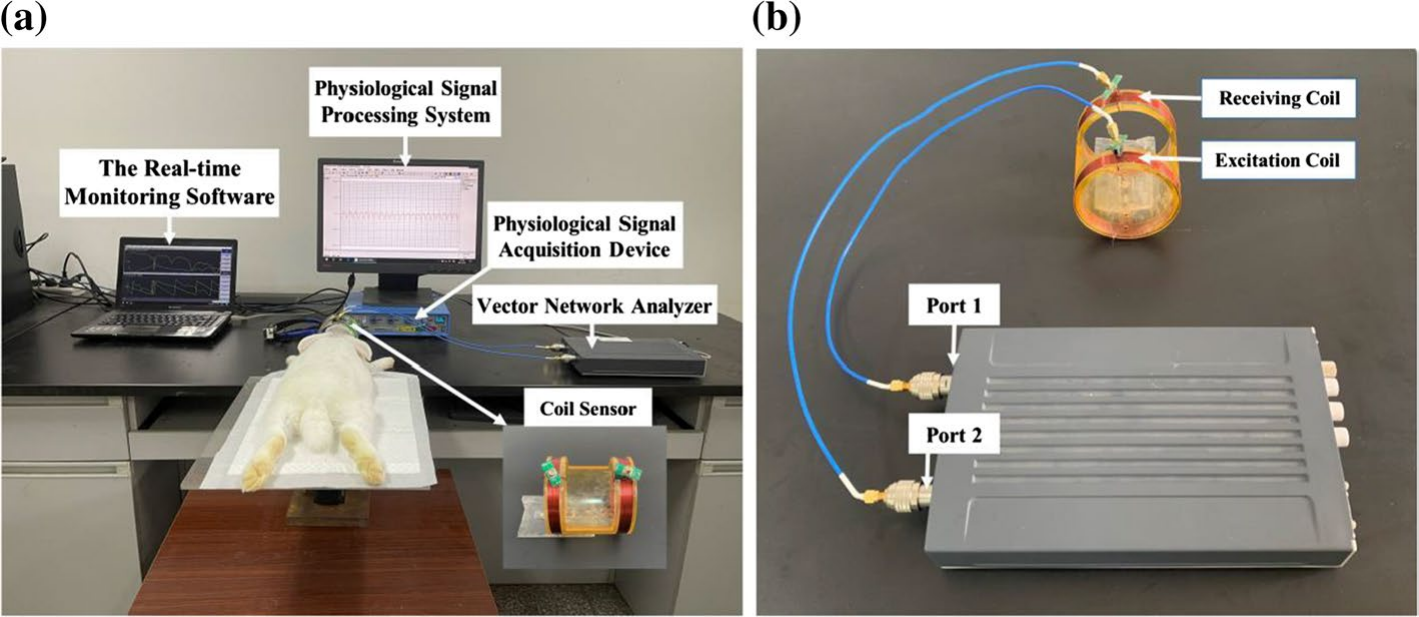
Experimental platform to monitor AIS in rabbits. It includes the multi-parameter near-field coupling sensing system and the multi-channel physiological signal acquisition and processing system (Chengdu Instrument Factory, RM6240XC). They are responsible for collecting the multiple NFC parameters and the respiratory frequencies. **a** represents the entire system. **b** shows the radiating part

In this study, the multi-parameter NFC measuring results of 16 rabbits before common carotid artery ligation were regarded as the control group. And the results of those after ligation were used as the experimental group. Moreover, the time difference offsetting was selected to remove partial environmental effects. The methods are as follows. (i) The multi-parameter NFC data of anesthetized rabbits were collected as referenced values. At this time, no other surgical operations were performed on the rabbits. (ii) After exposing and separating the bilateral common carotid arteries. the multi-parameter NFC data of the rabbits were measured. The differences between the data and the reference values were used as the control group without ischemia, corresponding to the zero-time phase of the monitoring results. (iii) The 6 h monitoring of multi-parameter NFC was conducted on the rabbits after ligation. The differences between the results and the reference values were regarded as the experimental group with ischemia, corresponding to the time phases from 1 to 6 h. As shown in Fig. 8, the rabbits were placed on the plexiglass plate equipped with the coil sensor, where their heads were placed in the geometric center of the coil sensor. The multi-channel physiological signal acquisition and processing system (Chengdu Instrument Factory, RM6240XC) was used to monitor the respiration of the rabbits. When the rabbits breathed normally, we ran the real-time monitoring software for AIS and started to collect RA, TA, RP and TP data synchronously. Meanwhile, the respiratory frequency was observed every one hour to ensure those rabbits in a stable state. After the experiment, the rabbits were euthanized by air injection method.

### Selection of feature frequency

**Fig. 9:**
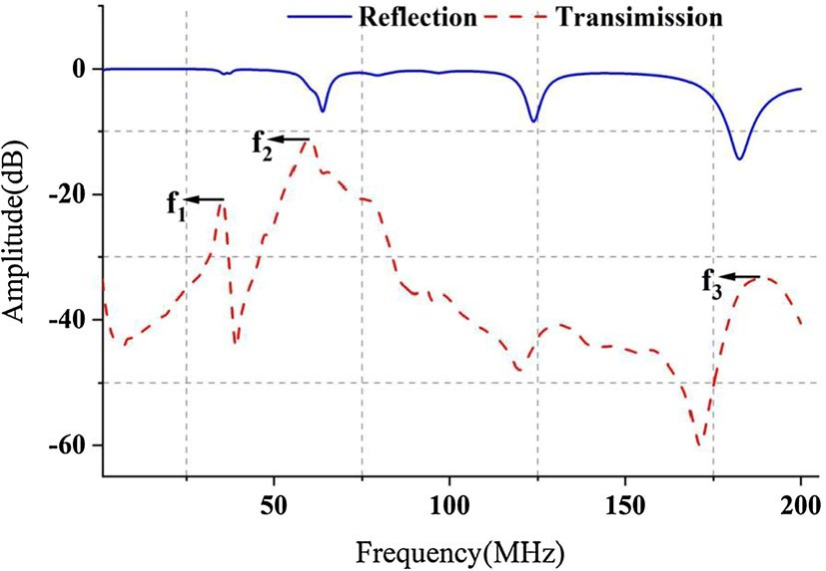
The initial average amplitude spectrum (*n* = 16) of the entire multi-parameter NFC sensing system containing rabbits in reflection and transmission. The frequency range is from 300 kHz to 200 MHz. In two-port test network, lower reflection amplitude (RA) represents less incident signal reflected back to port 1 and higher transmission insertion amplitude (TA) indicates more signals transmitted to port 2 through the brain

When rabbits were placed in the specified position of the coil sensor after anesthesia, the initial amplitude spectra of the entire multi-parameter NFC sensing system in reflection and transmission are shown in Fig. 9. In two-port test network, higher return loss represents less incident signals reflected back to Port 1 and lower insertion loss indicates more signals transmitted to Port 2 through the brain. With almost the same level of return loss, lower insertion loss shows higher transmission efficiency [46]. Based on this, three feature frequency points, f_1_(35.622 ± 0.219 MHz), f_2_(59.836 ± 0.179 MHz) and f_3_(189.141 ± 0.497 MHz) were found in sweeping. The f_2_ corresponded to the minimum insertion loss of the transmission signal, indicating that the impedance matching was optimal and the efficiency of the entire system was the highest. Under this condition, the four parameters (RA, TA, RP and TP) theoretically demonstrated strong stability and sensitivity. The f_1_ and f_3_ corresponded to the two frequency points with larger insertion loss of the transmission signal, indicating that most of the energy was lost in the signal transmission and reflection process, thus leading to worse performance in multi-parameter NFC sensing. Therefore, we proposed that the f_2_ was the optimal detection frequency of AIS, which would be proved by analyzing the changes of RA, TA, RP and TP in the sweeping and comparing the average variations in the FB around the three feature frequency points (FB_1_ = f_1_ ± 5 MHz, FB_2_ = f_2_ ± 5 MHz, FB_3_ = f_3_ ± 5 MHz).

f_1_(35.622 ± 0.219 MHz), f_2_(59.836 ± 0.179 MHz) and f_3_(189.141 ± 0.497 MHz) were regarded as three feature frequency points based on this.

### Statistical analysis

All the data are expressed as mean ± standard deviation from 16 independent experiments. The intra-group differences of four parameters in three periods (before ligation, 3 h after ligation and 6 h after ligation) were statistically analyzed by a paired-samples *t*-test to prove the effectiveness of multi-parameter NFC in distinguishing different pathophysiological states following AIS. The significance level was set at *α* = 0.05. Statistical analyses were performed by SPSS software version 22.0 (SPSS Inc., Chicago, USA).

### Classification of different ischemic states

The BP network, one of the most widely used artificial neural network, was selected for classification of ischemia at different degrees. Based on the change trends and the statistical results of the four parameters, the classification of three intracranial pathophysiological states was performed. Twelve groups of experimental data were randomly selected as training samples (*n* = 36), and the others were used as test samples (*n* = 12). In the process of identification, the four were used, respectively, or all together as recognition features, and the test samples were identified for three times. The MATLAB neural network toolbox was used to set up the model, the hidden layer node was set to 6 and the output layer node was set to 3. The hidden layer function was set as hyperbolic tangent sigmoid function and the output layer function was set as linear regression function. The training target was set to 0.00001. The output mean *P* values in three different intracranial pathophysiological states were based on the test samples corresponding to the state before classification (*n* = 4). What’s more, features were normalized before entering the neural network training.

## Data Availability

All data generated or analyzed during this study are included in this published article.

## Abbreviations

CBF: Cerebral blood flow
AIS: Acute ischemic stroke
NFC: Near-field coupling
BP: Back propagation
CT: Computed tomography
MRI: Magnetic resonance imaging
TCD: Transcranial Doppler
CBFV: Cerebral blood flow velocity
LDF: Laser Doppler flowmeter
NIRS: Near-infrared spectroscopy
BEI: Bioelectrical impedance
EW: Electromagnetic wave
TUT: Target under test
FB: Frequency band
RA: Reflection amplitude
TA: Transmission amplitude
RP: Reflection phase
TP: Transmission phase
IFBW: Intermediate frequency band width
